# The DUF582 Proteins of *Chlamydia trachomatis* Bind to Components of the ESCRT Machinery, Which Is Dispensable for Bacterial Growth *In vitro*

**DOI:** 10.3389/fcimb.2016.00123

**Published:** 2016-10-07

**Authors:** François Vromman, Stéphanie Perrinet, Lena Gehre, Agathe Subtil

**Affiliations:** ^1^Institut Pasteur, Unité de Biologie Cellulaire de l'Infection MicrobienneParis, France; ^2^CNRS UMR 3691Paris, France; ^3^Université Pierre et Marie Curie, Cellule Pasteur UPMCParis, France

**Keywords:** host pathogen interactions, *Chlamydia trachomatis*, type III effectors, ESCRT, Hrs, Tsg101

## Abstract

*Chlamydiae* are Gram negative bacteria that develop exclusively inside eukaryotic host cells, within a membrane-bounded compartment. Members of the family *Chlamydiaceae*, such as *Chlamydia trachomatis*, are pathogenic species infecting vertebrates. They have a very reduced genome and exploit the capacities of their host for their own development, mainly through the secretion of proteins tailored to interfere with eukaryotic processes, called effector proteins. All *Chlamydiaceae* possess genes coding for four to five effectors that share a domain of unknown function (DUF582). Here we show that four of these effectors, which represent the conserved set in all *Chlamydiaceae*, accumulate in the infectious form of *C. trachomatis*, and are therefore likely involved in an early step of the developmental cycle. The fifth member of the family, CT621, is specific to *C. trachomatis*, and is secreted during the growth phase. Using a two-hybrid screen in yeast we identified an interaction between the host protein Hrs and the DUF582, which we confirmed by co-immunoprecipitations in co-transfected mammalian cells. Furthermore, we provide biochemical evidence that a second domain of one of the DUF582 proteins, CT619, binds the host protein Tsg101. Hrs and Tsg101 are both implicated in a well conserved machinery of the eukaryotic cell called the ESCRT machinery, which is involved in several cellular processes requiring membrane constriction. Using RNA interference targeting proteins implicated at different stages of ESCRT-driven processes, or inhibition by expression of a dominant negative mutant of VPS4, we demonstrated that this machinery was dispensable for bacterial entry, multiplication and differentiation into infectious progeny, and for uptake of glycogen into the parasitophorous vacuole. In light of these observations we discuss how the DUF582 proteins might target the ESCRT machinery during infection.

## Introduction

Chlamydiae are obligate intracellular bacteria that grow in very diverse eukaryotic hosts, including humans. *Chlamydia trachomatis* is the most prevalent sexually transmitted bacterial pathogen. Infections of the urogenital mucosae often stay asymptomatic but can lead to severe pathologies including pelvic inflammatory disease, ectopic pregnancy, and infertility (Brunham and Rey-Ladino, 2005). This species is also able to colonize the eye conjunctiva, and the resulting inflammation is the leading cause of blindness by an infectious agent (Taylor et al., 2014).

All chlamydiae proliferate via an intracellular biphasic developmental cycle (AbdelRahman and Belland, 2005). The infectious forms of the bacteria, called elementary bodies (EBs), are small and non-replicative. Upon entry into a host cell, typically an epithelial cell, the EB converts to a larger, metabolically more active and replicative form, the reticulate body (RB) (Cossé et al., 2016). EBs and RBs reside within a membrane-bound vacuole called the inclusion. After several rounds of division, RBs convert back to the infectious form, before ultimately exiting the host cell. Completion of the whole cycle takes 2 or more days depending on the species.

*C. trachomatis* displays a genome reduced to around one million base pairs, and relies on the host with regard to several essential metabolic pathways (Stephens et al., 1998). Lipid droplets and peroxisomes have been observed in the inclusion lumen, indicating that this compartment is able to engulf large particles (Kumar et al., 2006; Boncompain et al., 2014). Also, we have recently shown that *C. trachomatis* is able to engulf glycogen in bulk from the host cytoplasm (Gehre et al., 2016). One piece of evidence for bulk import of cytoplasmic glycogen was the observation of glycogen-filled vesicles in the inclusion lumen, suggesting that the polymer was engulfed in a membrane-bound form, through inward invagination of the inclusion membrane. Similarly, live microscopy on the import of lipid droplets suggested that the inclusion membrane was able to engulf such large particles (Cocchiaro et al., 2008). The underlying mechanism is completely unknown. Topologically speaking, it is similar to the inward invagination of the limiting membrane of endosomes that leads to the formation of multivesicular bodies (MVBs), a well-studied step along endosomal maturation. The formation of luminal vesicles in MVBs depends on a machinery called the endosomal sorting complex required for transport, or ESCRT (Hurley, 2010; Field et al., 2011). During MVB biogenesis five distinct complexes (ESCRTs -0, -I, -II, and -III, and VPS4) act sequencially to recognize and sort ubiquitinated cargo into intraluminal vesicles (Henne et al., 2011). In addition to their role in MVB formation, for which they were first described, ESCRT proteins are well established to function in cell abscission, viral budding, exosome secretion, and autophagy (McCullough et al., 2013). The very ancient emergence of some of the ESCRT components explains the implication of this machinery in several core functions of the eukaryotic cell (Field et al., 2011; Hurley, 2015). Considering the fact that parasitism of eukaryotic cells by *Chlamydiae* is also a very ancient event (Horn et al., 2004), the bacteria may have acquired mechanisms to co-opt this machinery to sustain its own development.

Chlamydiae devote a large proportion of their small genome to the synthesis of proteins secreted into the host cytoplasm. By doing so, they hijack key cellular pathways and host resources for their own need. Among its different secretion machineries chlamydiae have privileged the type 3 secretion system for the translocation of proteins into the inclusion lumen and into the cytoplasm (Subtil et al., 2005; da Cunha et al., 2014; Mueller et al., 2014). In particular we have identified, as type 3 secretion substrates, a family of proteins conserved in all pathogenic strains with a common domain numbered DUF582 (Muschiol et al., 2011). The *C. trachomatis* genome encodes five DUF582 proteins, CT619, CT620, CT621, CT711, and CT712, that are expressed at mid- to late-cycle (Belland et al., 2003). CT620 and CT621 were detected in the host cytoplasm, and CT620 and CT711 were also detected in the nuclei of infected cells (Muschiol et al., 2011). The follow-up work described in the present paper pointed to the ESCRT-0 component Hrs as a potential partner. Hrs, together with its partner STAM, forms the ESCRT-0 complex. Both proteins bind ubiquitinated cargo. Through this activity, and their ability to bind early endosomes, they control the first step of sorting of ubiquitinated cargo toward intraluminal vesicles. Hrs also makes the link with the second step of cargo sorting through its ability to bind one subunit of the ESCRT-I complex, Tsg101.

The potential interaction between one DUF582 domain and Hrs, together with the attractive hypothesis that the ESCRT machinery might be involved in the uptake of cytoplasmic material by the inclusion, prompted us to investigate whether the ESCRT machinery was required for chlamydial development.

## Materials and methods

### Cells and bacteria

HeLa cells and HEK-293 cells (ATCC) were cultured in Dulbecco's modified Eagle's medium with Glutamax (DMEM, Invitrogen), supplemented with 10% (v/v) fetal bovine serum (FBS). *C. trachomatis* LGV serovar L2 strain 434 (ATCC), or GFP-expressing L2 (^GFP^CtrL2) (Vromman et al., 2014) were purified on density gradients as previously described (Scidmore, 2005).

### Yeast-two-hybrid (Y2H) assays

The Y2H screens were performed by Hybrigenics (Paris, France). Two baits were used corresponding to the DUF582 domain of CT619 (CT619_Cter_, amino acids 480–877) and to the N-terminal domain (amino-acids 1–490). For each screen, a minimum of 7.10^7^ interactions between the bait and a human placenta library were tested.

Interactions were further tested using the Matchmaker kit (Clontech) following the manufacturer's guidelines. The DUF582 protein constructs were cloned into the yeast vector pGBKT7 carrying the GAL4 DNA binding domain while the Hrs constructs were cloned into the pGADT7 vector carrying the GAL4 activation domain. The primers used to make these constructs are listed in the Table S1. The yeast strain AH109 was transformed with the two vectors simultaneously and plated on double dropout medium (DDO; SD/-Leu/-Trp) for 48 h at 30°C. Single colonies were then cultured over-night at 30°C in a YPD medium and from this culture a serial dilution of the same number of yeast was plated on selective media DDO and QDO (SD/-Ade/-His/-Leu/-Trp). Results were analyzed 48 h later.

### Transfections of plasmids or siRNA

Cells were transfected with the indicated plasmids 24 h after seeding using JetPrime transfection kit (Polyplus transfection) following the manufacturer's instructions. For gene silencing, 10 nM (final concentration) of siRNA (Dharmacon) was mixed with Opti-MEM (Invitrogen) and Lipofectamine RNAiMAX reagents (Invitrogen) and added to 80,000 HeLa cells in suspension in complete medium. The cells were subsequently seeded in 24-well plates. Transfection was performed twice, 48 and 4 h prior to infection. siRNA efficiency was determined by immunoblot or RT-PCR (see respective sections for details). The siRNA sequences used were the following: control UGGUUUACAUGU CGACUAA, Hrs (1) GAACCCACACGUCGCCUUG, Hrs (2) GAGGUAAAC GUCCGUAACA, Tsg101 (1) CCAGUCUUCUCUCGUCCUA, Tsg101 (2) GAA GUAGCCGAGGUUGAUA, Chmp4B AGAAAGAAGAGGAGGACGA (Guizetti et al., 2011).

### Cloning procedures

The genes coding the five DUF582 proteins (CT619, CT620, CT621, CT711, CT712) were amplified from *C. trachomatis* D/UW-3/CX genomic DNA by PCR with Phusion high-fidelity DNA polymerase (Finnzyme) according to the manufacturer's instructions and cloned into a pEGFP-derived destination vector providing a N-terminal green fluorescent protein (GFP) tag using the Gateway technology. The primers used to make these constructs are listed in the Table S1. Other constructs were generously provided by F. Gesbert (Université Paris Sud, Villejuif, France, myc-Hrs), E.O Freed (NIH Bethesda, USA, HA-Tsg101 constructs; Goila-Gaur et al., 2003), J. Martin-Serrano (King's College London School of Medicine, London, UK, VPS4 constructs).

### Production and purification of recombinant protein for antibody production

The full length gene of *ct712* and the region of *ct619* truncated of the first 81 and last 625 codons were amplified by PCR, and were cloned using the Gateway system into the pDEST15 (Invitrogen) destination vector, providing a GST tag at the N-terminus. Expression of the recombinant proteins was made after transformation of BL21 *E. coli* strain. BL21 bacteria transformed with the GST-Δ81CT619Δ625 construct were cultured in LB media supplemented with ampicillin at 37°C until the optical density at 600 nm reached 0.6 before addition of isopropyl β-D-1-thiogalactopyranoside (IPTG) for expression induction. The GST-CT712 construct was obtained from cultures in micro-fermentors to overcome low yields. Protein purification was performed on column using glutathione-sepharose beads (GE Healthcare) following the manufacturer indications. Purified proteins were used to immunize New Zealand White rabbits for production of polyclonal antisera (AgroBio, La Ferté Saint-Aubin, France). To test these antibodies by western blot, cell lysates or purified EBs were lysed in 8 M urea, 1% SDS (v/v), 150 mM NaCl, 30 mM Tris pH 8.0 and the proteins were analyzed by SDS-PAGE as described below.

### Immunofluorescence and western blot

For immunofluorescence (IF), HeLa cells fixed for 30 min at room temperature in paraformaldehyde (PFA) 4% (w/v) in PBS were permeabilized for 15 min in PBS supplemented with 1 mg/ml bovine serum albumin (BSA) and 0.05% (w/v) saponin (IF buffer). For anti-Hrs staining, cells were fixed for 30 min on ice in PFA 4%. For CT813 staining, cells were fixed 30 min at room temperature in PFA 2%. Coverslips were incubated with primary antibodies in IF buffer for 1 h at room temperature, followed with three washes in the same buffer. Primary antibodies used for IF were: mouse Anti-Hrs (clone A-5, Enzo Life Science), rabbit anti-Myc (ab9106, Abcam), rabbit anti-CT529 (Gehre et al., 2016), mouse anti-CT813 (kind gift from G. Zhong, University of Texas), rabbit anti-Gys1 (04-357, Millipore). Secondary antibodies coupled with a fluorophore were incubated for 1 h together with 0.5 μg/ml Hoechst 33342 (Molecular Probes) in IF buffer. Coverslips were then mounted in Mowiol buffer and analyzed using an Axio observer Z1 microscope equipped with an ApoTome module (Zeiss, Germany) and a 63 × Apochromat lens. Pictures were taken with a Coolsnap HQ camera (Photometrics, Tucson, AZ) using the software Axiovision.

For western blot analyses, cells were lysed in 1% (v/v) sodium dodecyl sulfate (SDS), 8 M urea, 150 mM NaCl, and 30 mM Tris-HCl pH 8. Samples normalized to protein content were analyzed by sodium dodecyl-sulfate poly-acrylamide gel electrophoresis (SDS-PAGE). Proteins were transferred on polyvinylidene difluoride (PVDF) membranes and incubated for 1 h in 0.1% Tween-20 (v/v) in PBS supplemented with 5% (w/v) skimmed milk. Primary and secondary antibodies were both sequentially incubated in 0.1% Tween-20 in PBS 1X on the membranes for 1 h separated by washes. Primary antibodies used were: rabbit anti-Hrs (Bethyl laboratories), mouse anti-Tsg101 (clone 4A10, GeneTex), anti-GFP (sc-8334, Santa-Cruz), and anti-HA.11 (clone 16B12, Covance), mouse anti-myc (clone 9E10, Santa-Cruz). For qualitative analysis, secondary antibodies were coupled with horse-radish peroxidase and were revealed by chemiluminescence (KODAK).

### Quantification of bacterial entry

Entry experiments were performed as described (Vromman et al., 2014). Briefly, HeLa cells were incubated at 4°C for 15 min in DMEM 10% FCS (v/v) before adding the ^GFP^CtrL2 (multiplicity of infection (MOI) = 10) for another 30 min at 4°C. Medium was replaced by pre-warmed medium at 37°C, and plates were transferred to the 37°C incubator for the indicated times before fixation in ice-cold 4% PFA in PBS for 30 min. Extracellular bacteria were stained with a mouse anti-MOMP-LPS (Argene #11-114) antibody followed with Cy5-conjugated secondary antibodies. Pictures of fields with 5-10 cells were acquired and analyzed as described (Vromman et al., 2014).

### Analysis of infection by flow cytometry

Cells were infected with density gradient purified ^GFP^CtrL2 EBs at a MOI < 0.3. Twenty-four hours later, cells were washed with PBS and gently detached using 0.5 mM EDTA in PBS. Samples were fixed in PFA 2% in PBS and stored over-night at 4°C. Flow cytometry analysis was performed with a FACS Gallios (Beckton Coulter) using the FL-1 (detecting fluorescence emission between 505 and 545 nm), the FSC (relative cell size) and the side scatter detectors (cell granulometry or internal complexity) on 1/10 of the sample diluted in PBS. A minimum total of 10,000 gated events were collected for each sample. Data were analyzed using the Kaluza 1.2 software (Beckman Coulter).

The analysis of cells transfected with myc-tagged VPS4 was performed in the following way: after fixation, the cells were centrifuged at 1500 g, washed in PBS, centrifuged again, and incubated for 1 h with rabbit anti-myc antibodies in 1 mg/ml BSA, 0.05% saponin in PBS. The cells were then washed and incubated for 1 h in the same buffer with anti-rabbit antibodies conjugated to Cy5. After another washing step the cells were resuspended in PBS and analyzed by flow cytometry in the FL-1 (green) and FL-4 (far-red) channels.

To measure progeny, cells were washed in PBS 24 h post-infection (hpi), detached and lysed with 1 mm glass beads. Fresh HeLa cells were inoculated with serial dilutions of the cell lysates and 24 h later flow cytometry was used to determine the infection rate and deduce inclusion forming units (ifu) in the inoculum.

### Immunoprecipitation (IP)

HEK-293 cells were lysed on ice for 45 min in ice cold 150 mM NaCl, 10 mM NaF, 1 mM EDTA, 1 mM EGTA, 0.5% Triton X-100 (v/v), 50 mM Tris pH 7.5 (IP buffer) supplemented with 5 mM PMSF, 1 mM vanadate and 1:100 of proteases inhibitor cocktail (P8340, Sigma). Cells were removed with a scraper, homogenized and centrifuged at 16000 *g* for 20 min at 4°C. For anti-myc IP, the supernatant was incubated at 4°C for 1–2 h with anti-myc 9E10 antibody (Santa-Cruz) followed by a 1.5 h incubation with protein G coupled to sepharose-beads. For anti-HA IP, the supernatant was incubated for 2 h with the anti-HA antibody coupled to agarose beads (Sigma) at 4 C. Immunoprecipitates were then washed at 4°C for a minimum of 5 times with 1 mL of the IP buffer with gentle centrifugations of maximum 400 g. Beads were then resuspended in a Laemmli buffer supplemented with 1% ß-mercapto-ethanol (v/v).

### Reverse transcription PCR

Total RNA was isolated with the RNeasy Mini Kit with DNase treatment (Qiagen) according to the manufacturer's protocol at the indicated times after transfection with control or Chmp4B siRNA. RNA concentrations were determined by NanoDrop and the samples normalized to an equal RNA content. Reverse transcription (RT) was performed with SuperScript III Reverse Transcriptase (Life Technologies) and cDNA was used as a template for amplification of Chmp4B with PrimeStar (Clontech) using GAGTTCCTGGAGAAGAAAATCG and CCTCTTCTTACGCTTCAGTGC as primers. Equal volumes were loaded on agarose gels and bands were revealed using UV-light visualizing ethidium bromide.

## Results

### The DUF582 domain interacts with Hrs

**Figure 1:**
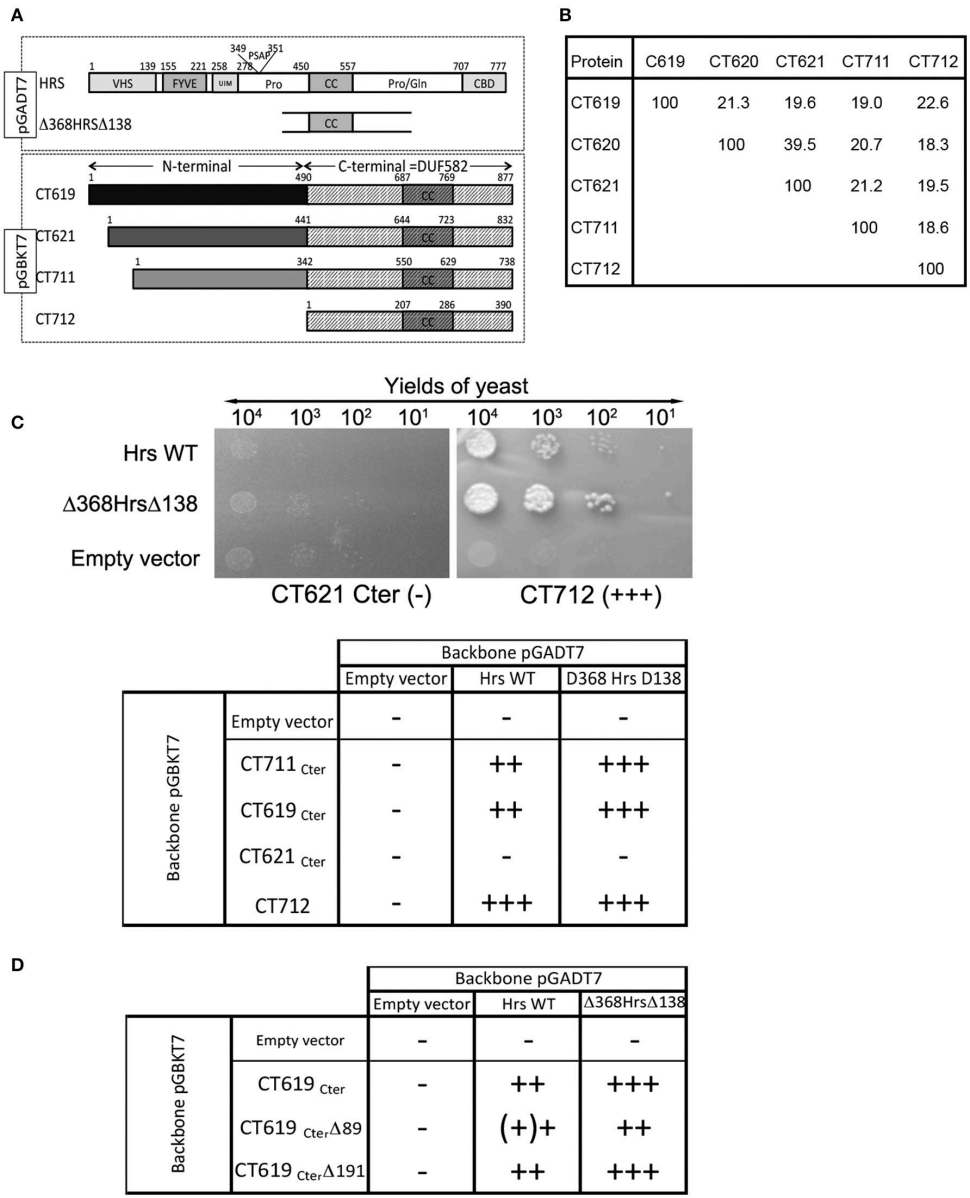
**Interaction between the DUF582 and Hrs probed by two-hybrid in yeast. (A)** Schematic view of the constructs used. Full length Hrs, or the minimal domain interacting with CT619 identified in the two-hybrid screen, Δ368HrsΔ138, were cloned in the pGADT7 vector. For the four chlamydial proteins tested, only the corresponding DUF582 (hatched box) were cloned in the pGBKT7 vector. **(B)** Percentage of identity between amino acids within the DUF582 domain of *C. trachomatis* DUF582 proteins **(C)** Illustration of one negative (CT621_Cter_) and one positive (CT712) interaction, and summary of the yields of yeast observed in the different combinations tested. **(D)** Summary of the yields of yeast observed when the last 89 or 191 amino acids of CT619_Cter_ were deleted.

A two-hybrid screen in yeast using the DUF582 domain of CT619 as bait identified 10 putative partners, with moderate to high scores of confidence. The best score was assigned to only two proteins: dynamin 2 and Hrs. Attempts to detect an interaction between CT619 and dynamin 2 by co-immunoprecipitation failed, and this direction was not pursued any further. The DUF582 domain is predicted to be mainly α-helical and to contain a segment adopting a coiled-coil conformation. It is only moderately conserved, with an amino acid identity ranging from 18% (CT711–CT712) to 39% (CT620-CT621) (Figure 1B and Muschiol et al., 2011). We reasoned that if, in spite of this low conservation, we could detect an interaction between Hrs and the DUF582 domain of other members of the family, this would considerably strengthen the hypothesis that Hrs is a true interactor of the domain. To test this, we fused the DUF582 domains of CT619, CT621, CT711, and CT712 of *C. trachomatis* L2 to the Gal4 binding domain coded by the pGBKT7 vector (CT620 was not tested in this assay). Full-length Hrs, or its minimal interacting domain found in the screen using the DUF582 of CT619 as bait, Δ368HrsΔ138, were fused to the Gal4 activation domain coded by the vector pGADT7 (Figure 1A). Yeast were co-transformed with each plasmid and plated on selective medium for isolation of co-transformed cells. Dilutions of the same quantity of co-transformed yeast were plated on high stringency medium (quadruple dropout) to reveal positive interactions (Figure 1C). All of the DUF582 domains tested, except that of CT621, showed an interaction with Hrs in this experimental system. For all cases where an interaction was observed, it appeared to be stronger when the shorter form of Hrs, Δ368HrsΔ138, rather than the full-length form, was tested.

To delimit further the interaction between CT619 and Hrs, we tested truncated versions of the DUF582 domain of CT619. We generated the construct CT619_Cter_Δ89, in which the last 89 amino acids, that correspond to the most conserved region in the DUF582 (Muschiol et al., 2011), were deleted. We also designed a construct truncated for the last 191 amino acids (CT619_Cter_ Δ191), which no longer contains the coiled coil region common to all DUF582 (Muschiol et al., 2011). Surprisingly, none of these truncations had an impact on the interaction with Hrs (Figure 1D). Thus, we concluded from these experiments that the ability to interact with Hrs is conserved between several DUF582 domains and does not involve the C-terminal part of this domain.

**Figure 2:**
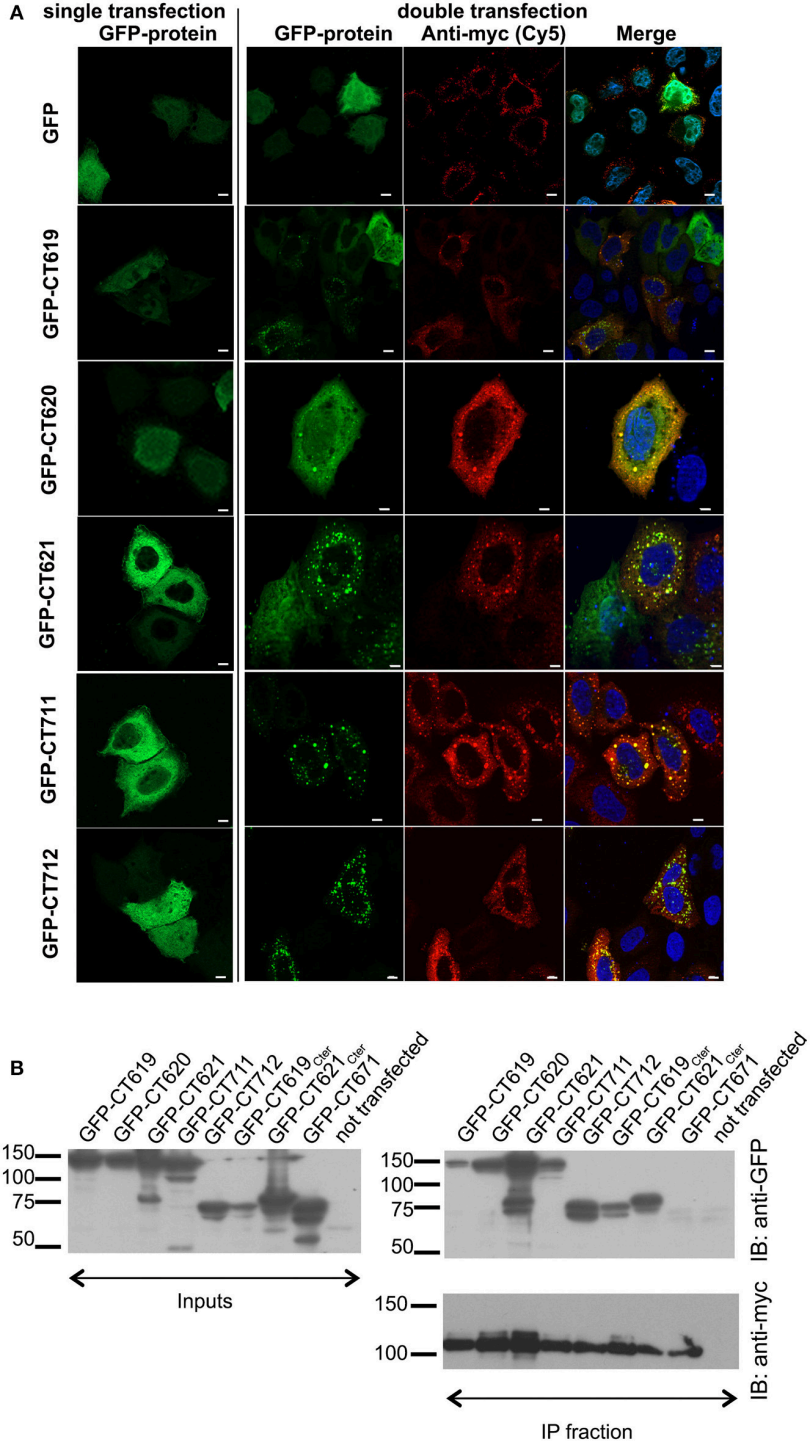
**Interaction between the DUF582 and Hrs probed by co-expression in mammalian cells. (A)** HeLa cells were transfected to express GFP alone or GFP-tagged DUF582 proteins, either alone (left) or together with myc-Hrs (right). One day later, the cells were fixed in 4% PFA and stained with rabbit anti-myc antibody, followed with a secondary Cy5-conjugated anti-rabbit antibody. DNA, labeled in blue, is shown on the merged picture. Scale bar = 10 μm. **(B)** HEK-293 cells were transfected with myc-Hrs and the indicated construct for 24 h, lysed and immunoprecipitated with anti-myc antibody. Proteins were separated on SDS-PAGE, transferred on a PVDF membrane and probed with the indicated antibody. An aliquot of each cell lysate was loaded on a separate gel to compare the expression level of each of the GFP-tagged proteins (input) and an aliquot of the immunoprecipitated fraction was loaded on a separate gel to assess the efficiency of the immunoprecipiation using anti-myc antibodies. IB, immunoblot; IP, immunoprecipitation.

To document this interaction with another technique we investigated the localization of GFP-tagged DUF582 proteins, in conditions where Hrs was overexpressed or not. When expressed alone, the GFP-tagged DUF582 proteins were uniformly distributed throughout the cytoplasm. When myc-Hrs was co-expressed, in the majority of the cells the distribution of the GFP-tagged proteins was no longer uniform, but accumulated in structures of various sizes (Figure 2A). The change in the distribution of the DUF582 proteins when Hrs was overexpressed, and their partial co-localization, support the hypothesis that the proteins interact. We tested this by co-immunoprecipitation experiments in HEK-293T cells overexpressing myc-Hrs together with various GFP-tagged proteins (Figure 2B). In these experiments a GFP-tagged fusion with an irrelevant chlamydial protein of similar size, GFP-CT671, was used as a negative control. No interaction was detected between this protein and myc-Hrs. In contrast, all the DUF582 proteins co-immunoprecipitated with myc-Hrs, including GFP-CT620, which had not been tested in the two-hybrid assay and colocalized with myc-Hrs. When only the DUF582 domain of CT619 was used, it was expressed at a lower level than the full-length protein and co-immunoprecipitated with myc-Hrs relatively better. Thus, it is possible that the DUF582 domain is somehow less accessible for Hrs binding when expressed in the full-length protein. Interestingly, we also detected an interaction between Hrs and CT621 or its DUF582 domain alone. It is consistent with the relocation of GFP-CT621 upon myc-Hrs co-expression reported above and indicates that, in spite of the negative result obtained in the two-hybrid screen, the DUF582 of CT621 is also able to bind the ESCRT-0 protein.

### CT619 also interacts with the ESCRT-I protein Tsg101

**Figure 3:**
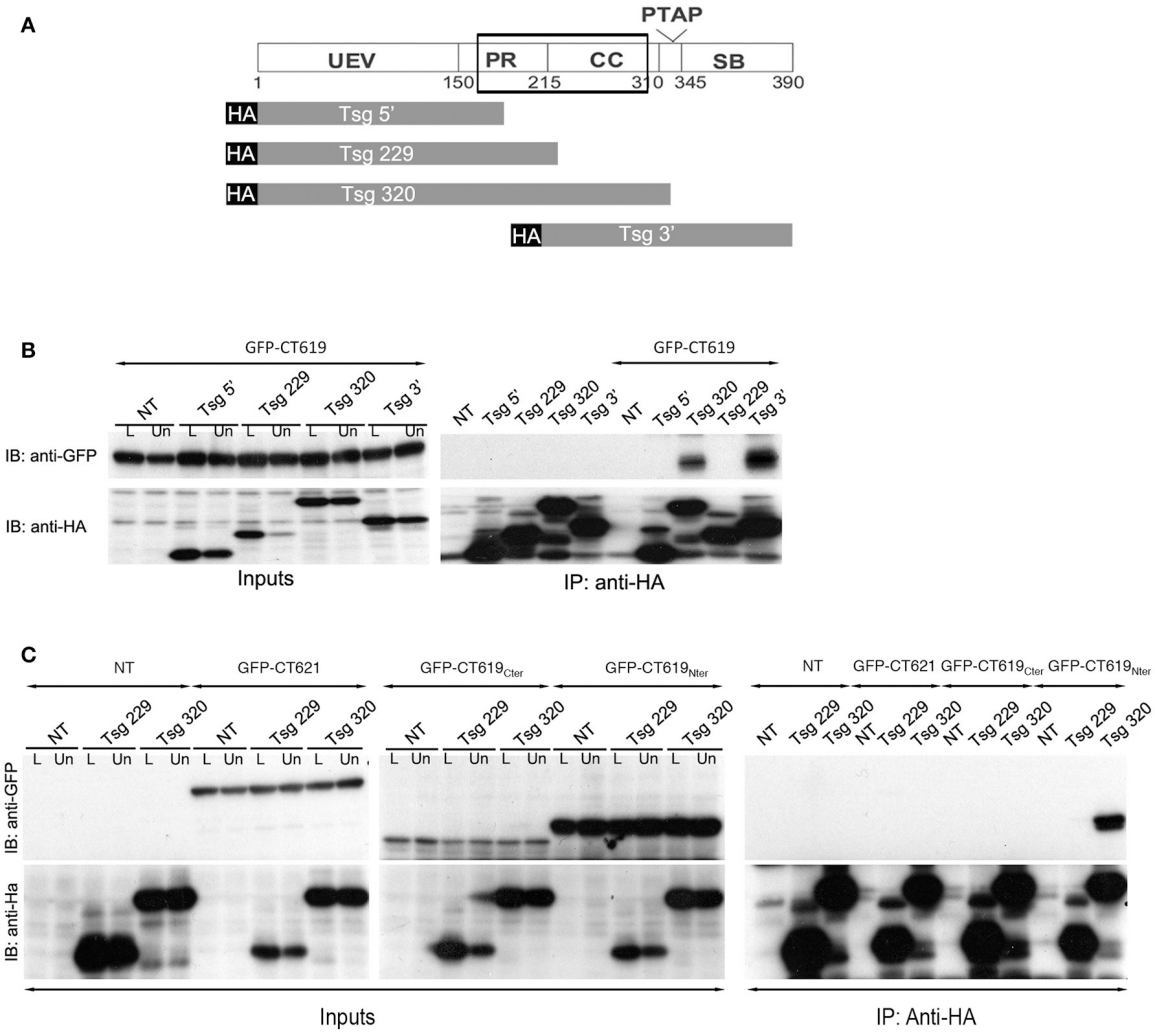
**The ESCRT-I protein Tsg101 interacts with CT619. (A)** Schematic view of the domains in Tsg101. The minimal region of interaction with CT619 identified in the two-hybrid screen is boxed. The four HA-tagged constructs used are schematized below. **(B)** HEK-293 cells were transfected with HA-tagged Tsg101 constructs alone or together with GFP-CT619 for 24 h, lysed and immunoprecipitated with anti-HA antibody coupled to agarose beads. Proteins were separated on SDS-PAGE, transferred on a PVDF membrane and probed with anti-GFP antibody. An aliquot of each cell lysate before (L) of after the immunoprecipitation (Un for Unbound) was loaded on a separate gel to compare the expression level of each of the proteins (Input, left, only shown for the co-transfections). IB, immunoblot; IP, immunoprecipitation. GFP-CT619 co-immunoprecipitated with the constructs Tsg320 and Tsg3', which include the coiled-coil domain of Tsg101. **(C)** Same as in B, with co-expression of HA-tagged Tsg101 constructs together with GFP-CT621, the DUF582 of CT619 (GFP–CT619_Cter_), or with its N-terminal domain (GFP-CT619_Nter_). Only the latter interacts with the Tsg320 construct.

A second two-hybrid screen in yeast, this time using the N-terminal domain of CT619 as bait, generated 20 hits with moderate to high confidence scores. Eleven proteins came out with the highest score: Bin3, Dact2, Dynamin-2, HMG20B, Keratins 8 and 19, PMC1, PHF16, RCN1, SPTB1, and Tsg101. The latter is a well-studied component of the ESCRT-I complex, and binds to Hrs. From the different clones obtained, that showed an interaction with the N-terminal domain of CT619, we inferred that the domain of interaction was comprised within the amino acids 168 and 282 of Tsg101, thus including parts of the proline-rich region and the coiled-coil domain (Figure 3A).

To verify this interaction, we performed immunoprecipitation experiments using truncated HA-tagged Tsg101 constructs (Figure 3A). HEK-293T cells were transfected with HA-tagged Tsg101 constructs alone or together with GFP-tagged CT619. Full-length HA-tagged Tsg101 was expressed at a very low level and was not included in these experiments. Immunoprecipitation was performed with anti-HA antibodies coupled to agarose beads. Inputs and immunoprecipitated fractions were analyzed by western blot with anti-HA and anti-GFP antibodies. CT619 co-immunoprecipitated with the two constructs of Tsg101 that contained the coiled-coil domain (Figure 3B). Using constructs that expressed separately the amino-terminal domain of CT619 (GFP-CT619_N__ter_) or its DUF582 domain (GFP-CT619_C__ter_), we confirmed that the interaction between Tsg101 and CT619 implicates the N-terminal domain of CT619. In contrast, CT621, whose N-terminal domain shows no similarity with the one of CT619 (Muschiol et al., 2011), does not interact with the Tsg101 construct containing the coiled-coil domain, nor with the shorter construct that was used as a negative control (Figure 3C).

Altogether, these data demonstrate specific interactions of the ESCRT-I protein Tsg101 and of the ESCRT-0 protein Hrs with the N-terminal and C-terminal domains of CT619, respectively, strengthening the hypothesis that CT619 targets an ESCRT-driven pathway.

### Depletion of Hrs or of Tsg101 does not affect *C. trachomatis* internalization

**Figure 4:**
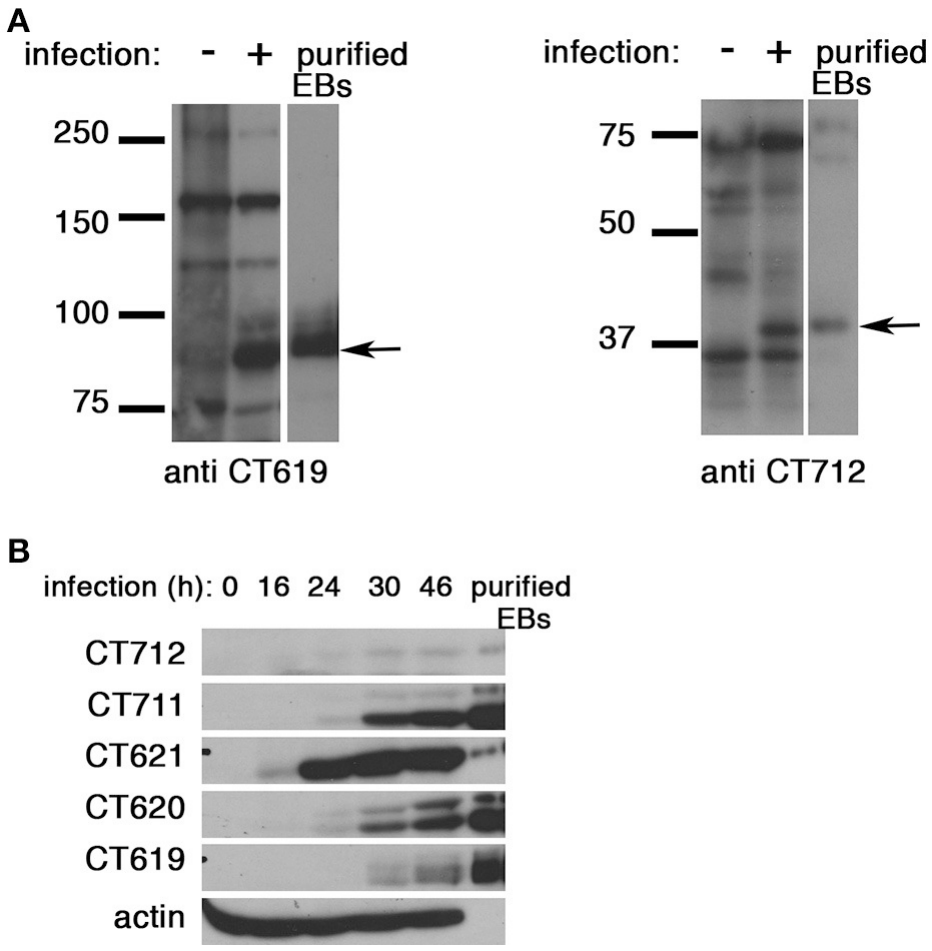
**All DUF582 proteins, except CT621, accumulate in EBs. (A)** Cell lysates from uninfected cells or cells infected for 32 h, and gradient purified EBs, were lysed in 8 M urea buffer (see methods). Proteins were separated on SDS-PAGE (10% for CT619, 12% for CT712), transferred on a PVDF membrane and probed with the indicated rabbit polyclonal antibody. CT619 (expected mw = 97 kD) runs as a double band, while CT712 (expected mw = 44 kD) runs as a single species. **(B)** HeLa cells were infected with *C. trachomatis* L2 for the indicated times before lysis in 8 M urea buffer. Gradient purified EBs, lysed in the same buffer, were loaded in the right lane for comparison. Identical samples were loaded on separate gels and probed with the indicated polyclonal rabbit antibodies. Actin was used as a loading control, and also shows the good purification of the EBs. CT712 expression is hardly detected. This might reflect a low expression, and/or sub-optimal detection with this antibody.

**Figure 5:**
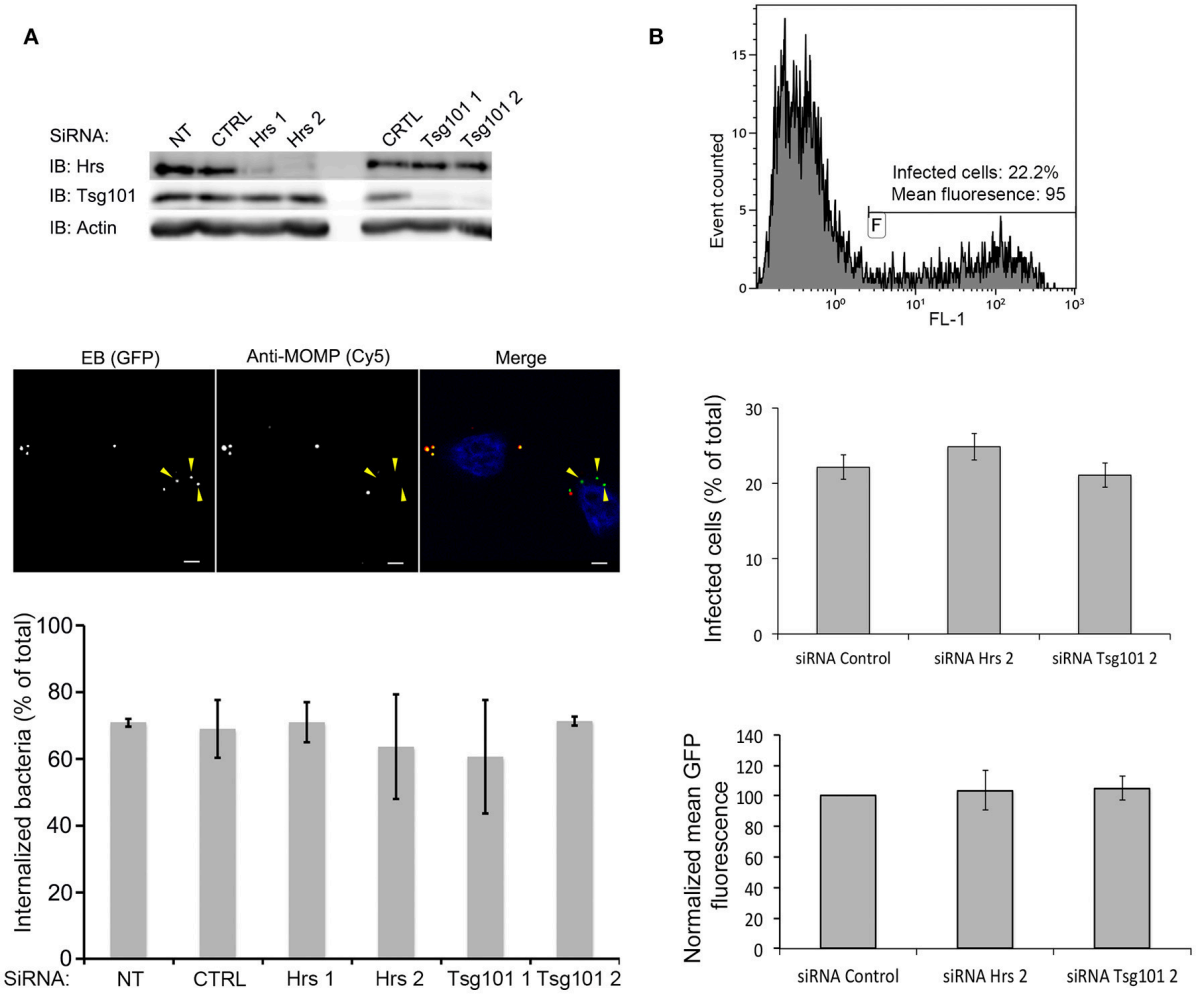
**Hrs and Tsg101 are dispensable for chlamydial entry and development**. HeLa cells were transfected for 48 h with siRNA targeting Hrs or Tsg101 (two different siRNA for each target) before infecting the cells with ^GFP^CtrL2. NT = not transfected, CTRL = transfected with control siRNA. **(A)** Sixty min after infection the cells were fixed, and extracellular bacteria were stained with anti-MOMP antibody, followed with Cy5-conjugated anti-mouse antibodies. The top panel shows the level of expression of Hrs and Tsg101 in cell lysates 24 h after the transfection revealed by western blot. Staining with antibodies against actin is shown as loading control. The middle panel depicts one representative field of the internalization assay, scale bar = 5 μm. Quantification of the internalized bacteria using the ICY software (Vromman et al., 2014) (>70 bacteria analyzed per condition) is presented in the histogram below. **(B)** Twenty-four h after infection the cells were fixed, and analyzed by flow cytometry. The top panel shows one representative sample: the level of green fluorescence (x axis, FL1) discriminates non-infected (left peak) from infected (right peak) cells. The middle panel shows the percentage of infected cells in the different conditions tested, and the lower panel shows the mean green fluorescence of the infected population. Values are the mean of three independent experiments, error bars represent the standard deviation.

To gain further insight into the function of the DUF582 proteins we completed our toolbox by generating polyclonal antibodies against CT619 and CT712 (Figure 4A). Like CT620 and CT711 (Muschiol et al., 2011), CT619 runs in denaturing gels as a double band, with the higher, minor, band corresponding to the expected molecular weight, and a 10 kD-shorter, major, species. CT712 migrates as a single species of expected size. CT619 and CT712 accumulated late in infected cells and were abundant in EBs (Figure 4B), a pattern of expression similar to that of CT711 and CT620. In contrast, CT621 was detected earlier (16 hpi) and was less abundant in EBs. Several type 3 secretion effectors are enriched in EBs (Saka et al., 2011), that are likely secreted in the early stages of infection, to facilitate entry and secure the nascent inclusion. By immunofluorescence, we did not detect any of the DUF582 proteins at an early stage of infection, possibly because the amount of effector secreted was too low for detection by this method (data not shown). At later time points we detected CT620 and CT621 in the host cytoplasm of some of the infected cells (Muschiol et al., 2011), but not the other DUF582 proteins (data not shown). Again, we cannot rule out the possibility that their secretion is below the sensitivity of this technique. Having shown that CT619 interacts *in vitro* with Hrs and Tsg101 we asked whether these proteins of the ESCRT machinery were required for the internalization step. Each of the two proteins was depleted for 24 h using two different siRNAs. Immunoblotting with specific antibodies revealed a depletion of at least 85% for each protein. Twenty-four h after depletion cells were infected with GFP-expressing *C. trachomatis* L2 (^GFP^CtrL2, MOI = 10), and fixed 1 h later. Extracellular bacteria were stained using anti-MOMP antibody and internalized bacteria were quantified using a semi-automated image analysis software (Vromman et al., 2014). No difference in the efficiency of entry was observed in the cells depleted for Hrs or Tsg101 compared to control cells (Figure 5A).

### *C. trachomatis* development proceeds normally *In vitro* in the absence of Hrs or Tsg101

By immunofluorescence we observed that Hrs was often present in the vicinity of the inclusion membrane, although it did not appear to be enriched at this location (Figure S1). It might transiently associate with the inclusion membrane and contribute to its exchanges with the host cytoplasm. We thus tested whether impairing ESCRT-driven pathways would impact bacterial growth. Control cells and cells depleted for Hrs or Tsg101 were infected with ^GFP^Ctr L2 at MOI = 1. Twenty h later cells were fixed and analyzed by flow cytometry (Vromman et al., 2014). Similar infection rates were observed in all samples, confirming that depleting Hrs or Tsg101 has no impact on bacterial entry (Figure 5B). In addition, GFP, a read-out of bacterial growth (Vromman et al., 2014), accumulated to the same extent in all conditions, indicating that Hrs and Tsg101 are not required for bacterial growth *in vitro* (Figure 5B).

### Disruption of late steps of the ESCRT machinery does not affect *C. trachomatis* development

**Figure 6:**
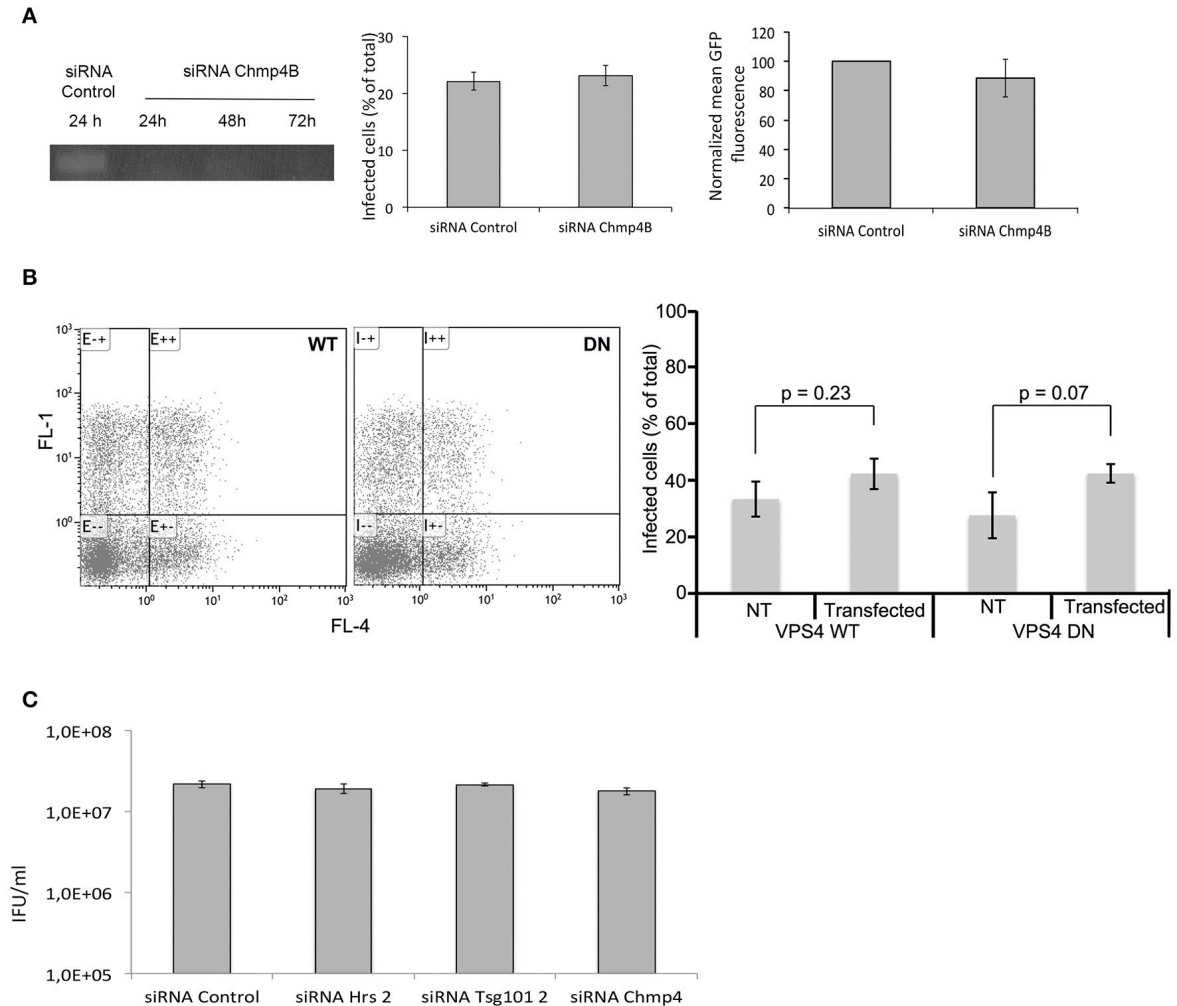
**ESCRT components are dispensable for completion of the developmental cycle *in vitro*. (A)** HeLa cells were transfected for 48 h with siRNA targeting Chmp4B or a control sequence before infecting the cells with ^GFP^CtrL2 and performing a second transfection of the same siRNA. Twenty-four h after infection the cells were fixed, and analyzed by flow cytometry. The left panel shows that the level of *Chmp4B* transcripts is strongly decreased as early as 24 h after the transfection with siRNA targeting this gene, and the depletion persists over the time of the experiment. The middle panel shows the percentage of infection, and the right panel shows the mean green fluorescence of the infected population. Values are the mean of three independent experiments, error bars represent the standard deviation. **(B)** HeLa cells were transfected with the WT or dominant negative (DN) form of myc-tagged VPS4, and infected with ^GFP^CtrL2 24 h later. Twenty-four h after infection the cells were fixed, permeabilized and stained with anti-myc antibody followed with Cy5-coupled secondary antibodies, and were analyzed by flow cytometry (Cy5 and GFP appear in the FL4 and FL1 channels respectively). One representative experiment is shown on the left, the average of 3 measurements is shown on the right (± standard deviation), *T*-test show no significant difference in the infections rates between transfected and non-transfected samples. **(C)** HeLa cells were transfected for 48 h with siRNA targeting Chmp4B or control sequence before infecting the cells with ^GFP^CtrL2. Twenty-four h after infection bacteria were collected and progeny was quantified in a reinfection assay. Values are the mean of three independent experiments (± standard deviation).

Hrs and Tsg101 are part of the ESCRT-0 and -I machineries, respectively, which sort cargo to the finale ESCRT-III driven step. Alternative adaptors appear to work upstream of ESCRT-III (Bissig and Gruenberg, 2014). Thus, to directly test if a functional ESCRT-III machinery was required for chlamydial growth we used a siRNA against Chmp4B, an essential component of the ESCRT-III complex (Hurley, 2010). The efficiency of the siRNA was verified by RT-PCR 24, 48, and 72 h post-transfection (Figure 6A). In addition, we examined the distribution of the epidermal growth factor (EGF) receptor using fluorescent EGF. Upon internalization with its ligand, the EGF receptor is processed by the ESCR-0, -I, -II, and -III machineries for finale sorting to multiversicular bodies (Henne et al., 2011). Depletion of ESCRT components alter the morphology of these compartments (Razi and Futter, 2006). Cells depleted for Chmp4 were loaded for 15 min with 100 ng/ml rhodamine-coupled EGF (EGF^Rho^), washed and incubated further at 37°C for 45 min before fixation. After treatment with siRNA against Chmp4B EGF^Rho^ positive compartments were fewer and more dispersed than in cell treated with control siRNA, confirming that this siRNA is efficient at disturbing Chmp4 mediated pathways (Figure S2). Cells were infected with ^GFP^CtrL2 at MOI = 1 24 h after the transfection with siRNA, and fixed and analyzed 24 h later by flow cytometry. Cells depleted for Chmp4B showed similar infection rates and bacterial load as control cells (Figure 6A), indicating that Chmp4B is not required for bacterial growth. Finally, to rule out the possibility that the levels of depletion obtained with the siRNA against Hrs, Tsg101, or Chmp4B, although clearly significant (see Figure S2 for functional validation of the siRNA against Hrs and Tsg101), were not sufficient to completely block ESCRT-mediated processes, we used a dominant negative form of VPS4, which prevents the disassembly of the ESCRT machinery at the end of the process, and is considered as the most potent method to inhibit ESCRT-mediated steps. Indeed the traffic of EGF^Rho^ was deeply affected by the expression of a dominant negative mutant of VPS4 (mutation K173 to Q) (Figure S2). HeLa cells were transfected with myc-tagged VPS4 constructs, wild type (WT) or dominant negative (DN), and infected 4 h later (to avoid excessive cell death upon expression of VPS4-DN). Cells were fixed 24 hpi and analyzed by flow cytometry. If anything, a slight increase in the percentage of infection was observed in the cells expressing myc-VPS4-DN compared to cells expressing the wild-type form. Bacterial loads were similar in both conditions (Figure 6B). This experiment confirms that functional ESCRT is not required for chlamydial growth.

Finally, we asked if impairing ESCRT-0, -I, or -III had an effect on the infectious progeny. We measured it on progeny collected 24 hpi, since prolonged (>72 h) depletion of the ESCRT proteins affected cellular viability in some of the experiments. Depletion of Hrs, Tsg101 or Chmp4B had no effect on the infectious progeny (Figure 6C), indicating that the whole infectious cycle proceeds normally in cells deficient for ESCRT functions.

### Import of cytosolic glycogen synthase is not dependent on the ESCRT machinery

**Figure 7:**
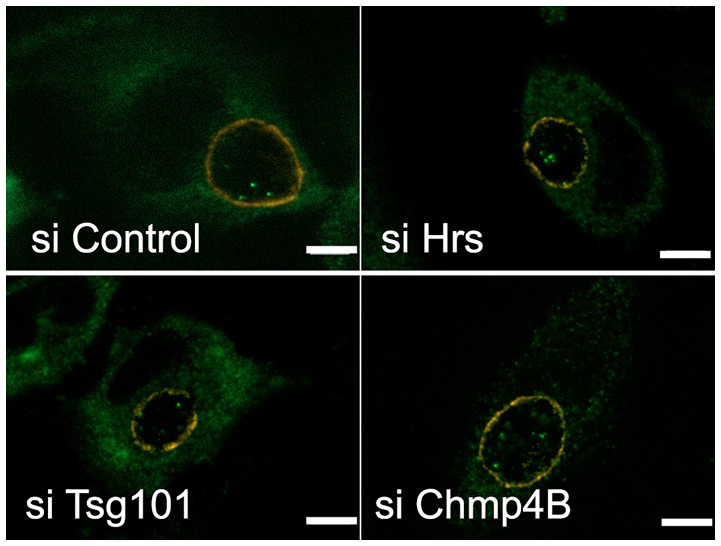
**Disruption of the ESCRT machinery does not impair the translocation of the host glycogen synthase into the inclusion lumen**. HeLa cells were transfected for 48 h with siRNA targeting the indicated targets before infecting the cells with CtrL2. Transfection was repeated 4 h after infection to ensure prolonged silencing of the targets. Cells were fixed 20 hpi in 2% PFA and permeabilized. The inclusion membrane was stained in red using antibodies against CT813, followed with Cy3-coupled anti-mouse secondary antibodies, and the host glycogen synthase was stained in green using rabbit antibodies followed with Alexa488-coupled secondary antibodies. Z-sections were taken throughout the volume of the inclusions and the pictures show one central slice in the inclusion volume. In all conditions examined Gys1 positive particles were observed inside the lumen of the inclusion. Scale bar = 5 μm.

We have recently shown that host glycogen translocates into the inclusion lumen, where it contributes to glycogen storage within the inclusion lumen. The host glycogen synthase Gys1 was also observed inside the inclusion lumen. This enzyme is known to associate with glycogen particles (Stapleton et al., 2010). When cytoplasmic glycogen levels were reduced by depriving the cells of glucose Gys1 translocation was also strongly reduced, supporting the hypothesis that Gys1 makes its way to the inclusion lumen bound to cytoplasmic glycogen (Gehre et al., 2016). The mechanism by which host glycogen/Gys1 reaches the inclusion lumen is unknown. By electron microscopy we observed glycogen-filled vesicles in the inclusion lumen, supporting a transport through the inward budding of vesicles from the inclusion membrane. Since this would fit with the topology of inward vesicle budding from the periphery of MVBs we hypothesized that the ESCRT machinery might be involved. To test this we silenced Hrs, Tsg101, or Chmp4B expression for 2 days before infecting the cells with *C. trachomatis* L2. Cells were fixed 24 h later, permeabilized, and the inclusion membrane and Gys1 were labeled with mouse anti-CT813 and rabbit anti-Gys1 antibodies, respectively. Strong reduction in Hrs, Tsg101, or Chmp4B levels did not impair Gys1 translocation into the inclusion lumen when compared to control cells (Figure 7), implying that the ESCRT-0, -I, and -III machineries are dispensable for this process.

## Discussion

The aim of the present work was to understand the function of a family of type 3 secretion substrates of *Chlamydiaceae*, which shared a domain restricted to this family, called DUF582. The domain showed only limited sequence identity between members of the family, and this feature incited us to use it as a lead for the functional study of the family. Using two-hybrid in yeast as a read-out for protein interaction we identified Hrs as a potential interactor for three DUF582 domains (those of CT619, CT711, and CT712). This finding was strengthened by the observation that co-expression of Hrs modified the distribution of each of the five DUF582 proteins of *C. trachomatis* when they were expressed in HeLa cells, and that the two proteins co-localized to some extent. In addition the DUF582 proteins co-immunoprecipitated with Hrs. Furthermore, the link between DUF582 proteins and the ESCRT pathway was strongly reinforced in the case of CT619, as the N-terminal domain of the protein interacted with a second protein of the pathway, Tsg101, both by two-hybrid and by co-immunoprecipitation in transfected cells.

In spite of this biochemical evidence for an interaction between the DUF582 domain and Hrs (and Tsg101 for CT619) we could not demonstrate its relevance in an infectious context. Three main limitations might explain the lack of direct evidence for a link between the DUF582 proteins and manipulation of the ESCRT machinery during infection.

First of all, the DUF582 proteins are of low abundance. Only CT619 and CT711 were detected in the first quantitative proteomic study performed on *C. trachomatis* (Saka et al., 2011). Recently, CT619, CT620, and CT621 were detected by proteomics, still within the low range in terms of molecules per bacterium (Skipp et al., 2016). Thus, it is unlikely that the secreted effectors are present in sufficient amount to globally disturb ESCRT-dependent processes, even more so when only a fraction of the pool might be translocated into the host cytoplasm at any given time. Indeed, we did not observe any major change in Hrs distribution during the infectious cycle, and we could not co-immunoprecipitate any of the DUF582 proteins together with Hrs in an infectious context.

Secondly, it is likely that the DUF582 proteins (with the exception of CT621, see below) are secreted at a very early step of infection, and are therefore present in minute amount in the host cytoplasm, challenging detection of the endogenous proteins and their activities. Indeed we demonstrated in this study using specific antibodies that CT619, CT620, CT711, and CT712 are enriched in EBs, consistent with a recent study of gene expression networks in *Chlamydiae* showing that the four genes are co-regulated (Domman and Horn, 2015). CT621, a *C. trachomatis* specific protein, is expressed earlier and is relatively less abundant in EBs. It is thus probably secreted only during the replication phase, a stage at which it was observed in the host cytoplasm (Muschiol et al., 2011). In contrast, it is likely that secretion of the four other DUF582 proteins starts just after the entry step, as for other pre-packed effectors (Cossé et al., 2016). We did not detect early secretion with our antibodies, likely because the proteins were not abundant enough. We generated a stable *C. trachomatis* L2 strain expressing CT619 fused to a C-terminal FLAG tag, but could only achieve a low expression of the protein, which was only detected in a few bacteria per inclusion, precluding its use in immuno-localization assays (data not shown). We occasionally observed endogenous Hrs in the immediate vicinity of internalized bacteria, supporting the hypothesis that an interaction between Hrs and DUF582 proteins might occur at an early step of the developmental cycle, possibly transiently. However, the frequency of these observations was too low to rule out that they occurred by chance, and further experimentation will be needed to test this hypothesis. Finally, it is unclear how the interaction between the DUF582 domain and Hrs relate to our previous detection of CT620 and CT711 in the nuclear fraction of infected cell (Muschiol et al., 2011), since to the best of our knowledge there is no evidence for nuclear location for Hrs.

Thirdly, as discussed below, we provide evidence here that the ESCRT machinery is not required for *C. trachomatis* development *in vitro*, indicating that rather than hijacking an ESCRT-driven host mechanism, the DUF582 domain might disable an ESCRT-driven function that would otherwise be detrimental to the bacteria. In that case, silencing ESCRT proteins would only reproduce what occurs normally, explaining the absence of phenotype on infection. An overexpression strategy cannot be used to test this hypothesis because overexpression of the ESCRT proteins disrupts most ESCRT-driven functions. Future work could consider generating strains knocked-out for one of the DUF582 proteins. We failed at generating CT619 deletion mutants, possibly because of technical shortcomings. In any case, the possible redundancy between the DUF582 proteins might require silencing of several genes simultaneously, a challenging prospect.

Thus, the relevance of the DUF582/ESCRT interaction in infection remains to be demonstrated. Future experimentation could consider the following hypotheses.

(i) Hypothesis 1: DUF582 proteins target Hrs/Tsg101 to escape lysosomal degradation. It is established that at least some of the receptors that chlamydiae use to bind to host cells are targeted to degradation after endocytosis. For instance *C. trachomatis* and *C. pneumoniae* enter the cell via the PGDF receptor and the EGF receptor, respectively (Elwell et al., 2008; Mölleken et al., 2013). Both receptors are degraded via the endo-lysosomal pathway, which is initiated by the Hrs recognition of the poly-ubiquitinated activated receptor. Bacterial effectors translocated upon invasion might reach a local concentration sufficient to bind Hrs (and Tsg101) and prevent the recruitment of the ESCRT machinery, thereby escaping from the lysosomal pathway. Such a restriction mechanism has been proposed for an effector of *Mycobacterium tuberculosis* (Mehra et al., 2013).

(ii) Hypothesis 2: DUF582 proteins target Hrs/Tsg101 to acquire material from the host. While *Chlamydia* imports many host constituents inside the inclusion, the mechanisms involved are largely unknown. Here we demonstrated that translocation of the host glycogen synthase Gys1 did not require the ESCRT machinery. Importantly, redundant pathways exist to sustain nutrient acquisition by the chlamydial inclusion (Vromman and Subtil, 2014). Thus, blocking one ESCRT-driven mechanism might not necessarily prevent uptake of one substrate, nor impact bacterial growth, making this second hypothesis compatible with our results.

(iii) Hypothesis 3: CT619 interacts with Tsg101 for bacterial exit through extrusion. *C. trachomatis* exits cells through two pathways, one being extrusion, that is budding of part of the inclusion surrounded with plasma membrane, without cell lysis (Hybiske and Stephens, 2007). Extrusion seems to have many similarities with cytokinesis, and ends by an abscission-like process. Cytokinesis requires Tsg101 activity, which is recruited before abscission to trigger ESCRT-III recruitment (Agromayor and Martin-Serrano, 2013). Proteins implicated in cytokinesis (i.e., MLCK and myosin II) have been found to be determinant for *Chlamydia* extrusion and their depletion decreases the number of extrusion events. Thus, secretion of CT619 late in the cycle could recruit Tsg101 to engage exit through the extrusion pathway. We have attempted to test this hypothesis but in our experimental system extrusion represents only a very minor pathway of cell exit, compared to bacterial lysis, precluding further analysis of the possible implication of Tsg101 and CT619 in this process.

Irrespective of the functional link between the DUF582 domain and Hrs, this study revealed that the ESCRT machinery was not required for *C. trachomatis* development *in vitro*. We showed that silencing of essential proteins of the ESCRT-0 (Hrs), -I (Tsg101), and -III (Chmp4B) machineries had no impact on primary infection, nor on the progeny collected. Consistently, overexpression of a dominant negative form of VPS4, an essential player of ESCRT driven processes, had no impact on bacterial growth. This result is surprising considering the implication of ESCRT machineries in a wealth of cellular processes (Hurley, 2015). It does not imply that ESCRT-driven processes are not co-opted by the bacteria, as proposed in the last two hypothesis discussed above. However, in a different perspective, silencing of major constituents of the ESCRT machinery must have deeply disturbed several cellular activities of the host. The fact that it had no impact on bacterial development speaks for the remarkable robustness of the bacterial development, which, while fully dependent on the host for many constituents, is able to adapt to deep variations in cell homeostasis.

## Author contributions

FV performed the experiments, interpreted the data, wrote the article; SP performed the experiments, interpreted the data, LG performed the experiments, interpreted the data, AS, conceived and designed the project, interpreted the data, wrote the article.

### Conflict of interest statement

The authors declare that the research was conducted in the absence of any commercial or financial relationships that could be construed as a potential conflict of interest.

## Supplementary material

Figure S1 **Distribution of Hrs during *C. trachomatis* development**. HeLa cells were infected with *C. trachomatis* L2 (MOI = 0.3) for the indicated times before fixation on ice in 4% PFA. The inclusion membrane was stained with anti-CT529 antibodies followed with Alexa488-conjugated anti-rabbit antibodies (green), Hrs was stained with mouse antibodies followed with Cy5-conjugated anti-mouse antibodies (red) and DNA was labeled with Hoechst 33342 (blue). Scale bar = 5 μm.

Figure S2 **Effect of the depletion of ESCRT proteins or of expression of VPS4 DN mutant on EGF^Rho^ traffic**. HeLa cells, transfected for 48 h with the indicated siRNA (top and middle panels) or for 24 h with VPS4 DN (bottom panels), were incubated for 15 min at 37°C with 100 ng/ml EGF^Rho^ in DMEM, washed, and incubated further for 45 min in complete medium before fixation in 4% PFA. Cells transfected with VPS4 DN were permeabilized and stained with Rabbit anti-myc antibodies followed with Alexa488-coupled anti-rabbit antibodies. For each condition one representative field in the red channel (EGF^Rho^) is shown. For the VPS4 DN transfection, expression of the VPS4 DN mutant is verified by anti-myc staining (bottom right panel). Scale bar = 10 μm. Note that the inhibition of the ESCRT components has distinct effects on the morphology of the EGF-containing compartments (Razi and Futter, 2006). It also affects the quantity of EGF receptor that recycles to the cell surface, and its degradation rate. Altogether, interfering with the activity of ESCRT components affects to various degree the intensity of the EGF^Rho^ signal (very reduced in Chmp4 siRNA treated cells or VPS4 DN expressing cells), and/or its distribution (scattering vs. clustering of the EGF^Rho^ positive compartments in Hrs and Tsg101 siRNA treated cells respectively).

Table S1 **Primers used in this study**.
