# Supplementary figures and images for: The DUF582 Proteins of Chlamydia trachomatis Bind to Components of the ESCRT Machinery, Which Is Dispensable for Bacterial Growth In vitro
Source: Front Cell Infect Microbiol. 2016 Oct 7;6:123. doi: 10.3389/fcimb.2016.00123 (PMC5053991; doi:10.3389/fcimb.2016.00123)

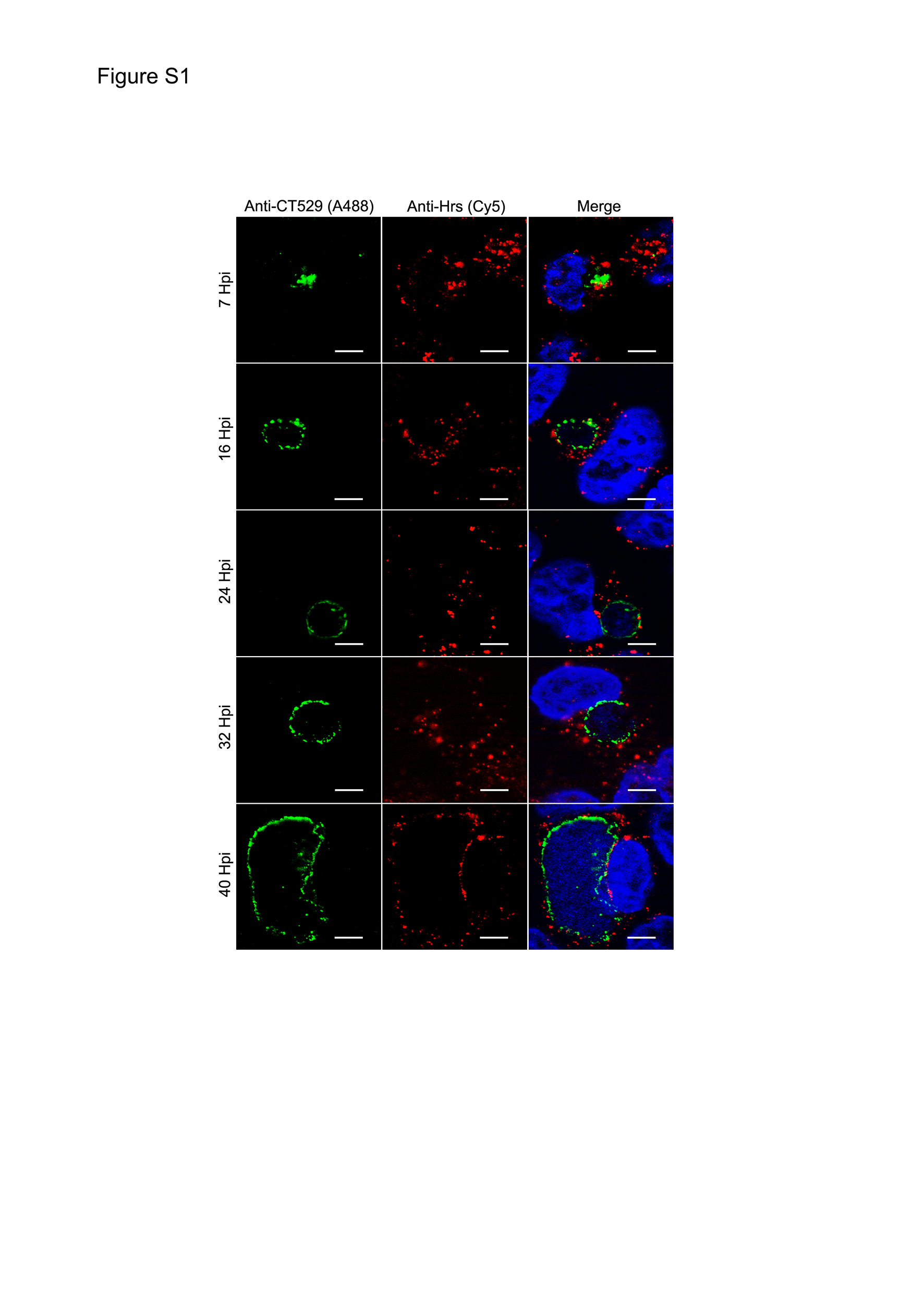

Supplement: Figure S1 — Distribution of Hrs during C. trachomatis development. HeLa cells were infected with C. trachomatis L2 (MOI = 0.3) for the indicated times before fixation on ice in 4% PFA. The inclusion membrane was stained with anti-CT529 antibodies followed with Alexa488-conjugated anti-rabbit antibodies (green), Hrs was stained with mouse antibodies followed with Cy5-conjugated anti-mouse antibodies (red) and DNA was labeled with Hoechst 33342 (blue). Scale bar = 5 μm. [file Image1.TIF]

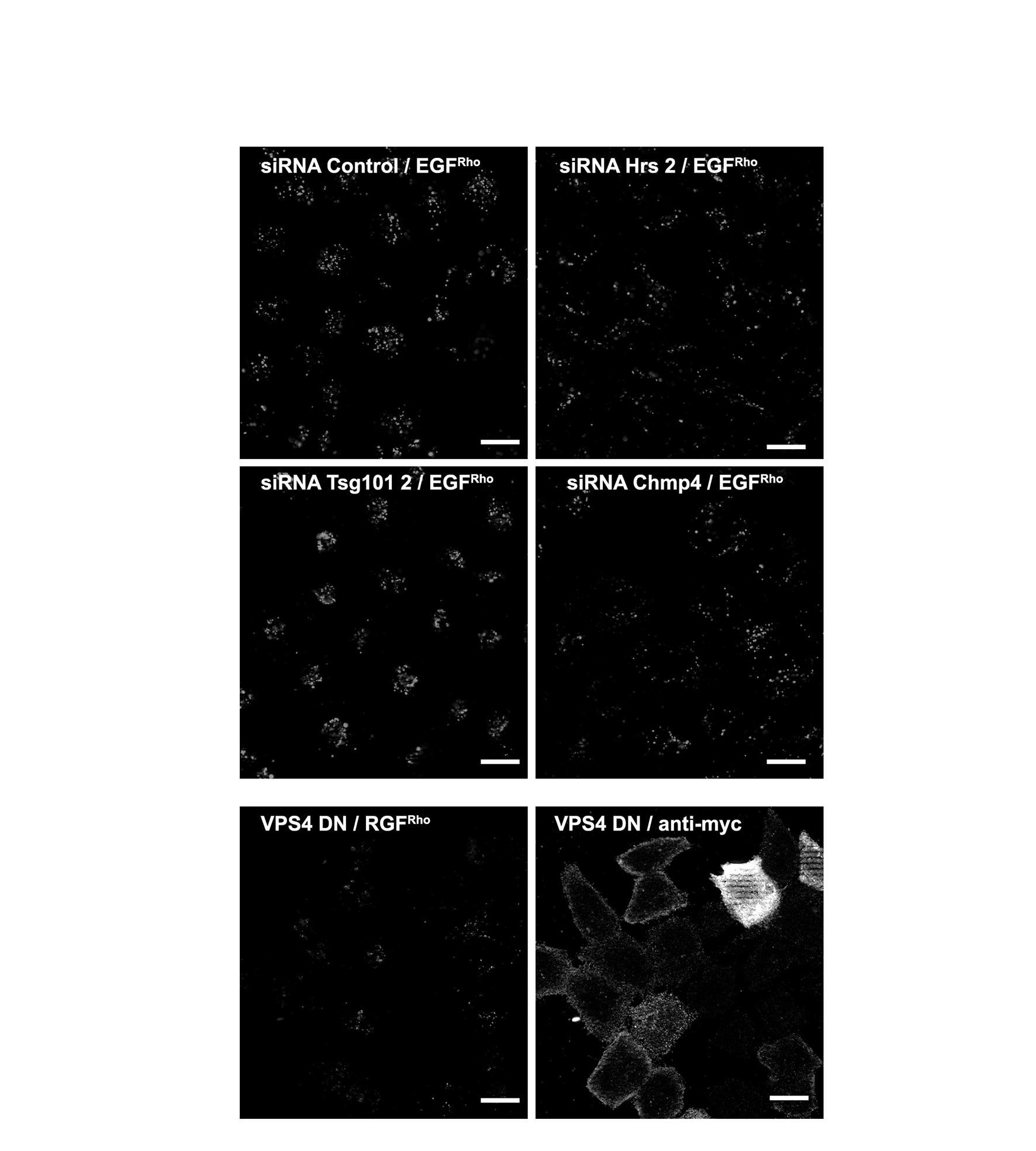

Supplement: Figure S2 — Effect of the depletion of ESCRT proteins or of expression of VPS4 DN mutant on EGFRho traffic. HeLa cells, transfected for 48 h with the indicated siRNA (top and middle panels) or for 24 h with VPS4 DN (bottom panels), were incubated for 15 min at 37°C with 100 ng/ml EGFRho in DMEM, washed, and incubated further for 45 min in complete medium before fixation in 4% PFA. Cells transfected with VPS4 DN were permeabilized and stained with Rabbit anti-myc antibodies followed with Alexa488-coupled anti-rabbit antibodies. For each condition one representative field in the red channel (EGFRho) is shown. For the VPS4 DN transfection, expression of the VPS4 DN mutant is verified by anti-myc staining (bottom right panel). Scale bar = 10 μm. Note that the inhibition of the ESCRT components has distinct effects on the morphology of the EGF-containing compartments (Razi and Futter, 2006). It also affects the quantity of EGF receptor that recycles to the cell surface, and its degradation rate. Altogether, interfering with the activity of ESCRT components affects to various degree the intensity of the EGFRho signal (very reduced in Chmp4 siRNA treated cells or VPS4 DN expressing cells), and/or its distribution (scattering vs. clustering of the EGFRho positive compartments in Hrs and Tsg101 siRNA treated cells respectively). [file Image2.TIF]
