# Supplementary material for: The DUF582 Proteins of Chlamydia trachomatis Bind to Components of the ESCRT Machinery, Which Is Dispensable for Bacterial Growth In vitro
Source: Front Cell Infect Microbiol. 2016 Oct 7;6:123. doi: 10.3389/fcimb.2016.00123 (PMC5053991; doi:10.3389/fcimb.2016.00123)
Supplement: Table S1 — Primers used in this study. [file Table1.DOCX]

Supplementary Table S1: List of primers used

Primers used for the two-hybrid in Yeast:

In pGBKT7 (GAL4 binding domain):

| **Construct** | **Primers** | |
| --- | --- | --- |
| CT619Cter | For | atgcggatccctgatgagaatacagtgattcaaaa |
|  | Rev | atgcctgcagttaattagagtaaatttccgctgcta |
| CT619Cter∆191 | For | atgcggatccctgatgagaatacagtgattcaaaa |
|  | Rev | atgcctgcagttaacaaaataagttttctccctcct |
| CT619Cter∆89 | For | atgcggatccctgatgagaatacagtgattcaaaa |
|  | Rev | atgcctgcagttaagttatcttatatttatccttgtcataattgt |
| CT621Cter | For | atgcggatccgtaataactttgttttgacggc |
|  | Rev | atgcctgcagctatcttaagagattacgcgctaatc |
| CT711Cter | For | atgcggatccataatgcacaagcttcttttacagatt |
|  | Rev | atgcctgcagttatttaaatctacggatcaacttagca |
| CT712 | For | atgcggatccatgcagaagtagcagccgactac |
|  | Rev | atgcctgcagctagctagaagccaatgttctatatacattat |

In pGADT7 (GAL4 activation domain):

| **Construct** | **Primers** | |
| --- | --- | --- |
| Hrs | For | atggatccatatggggcgaggcagc |
|  | Rev | gatcctcgagtcagtcgaatgaaatgagctg |
| ∆368Hrs∆138 | For | atggatccataccaacgtggtggagaacc |
|  | Rev | gatcctcgagtcatgctgggtacatgtaggcac |

List of primers used for Gateway cloning

| **Construct** | **Primers** | |
| --- | --- | --- |
| ∆81CT619∆625 | For | ggggacaagtttgtacaaaaaagcaggcttggaaggagatagaaccatggtaagaagagccgcaggag |
|  | Rev | ggggaccactttgtacaagaaagctgggtcctatcaaatcattttcaacacagaatcaat |
| CT619 | For | ggggacaagtttgtacaaaaaagcaggcttggaaggagatagaaccatggcttcttactatttaaattttaggccaact |
|  | Rev | ggggaccactttgtacaagaaagctgggtcctattaattagagtaaatttccgctgc |
| CT619_Cter_ (DUF582_CT619_) | For | ggggacaagtttgtacaaaaaagcaggcttggaaggagatagaacaatggtgatccaaaaagttagaaataaatgg |
|  | Rev | ggggaccactttgtacaagaaagctgggtcctattaattagagtaaatttccgctgc |
| CT619_Nter_ | For | ggggacaagtttgtacaaaaaaagcaggcttggaaggagatagaaccatggcttcttactatttaaattttaggccaact |
|  | Rev | ggggaccactttgtacaagaaagctgggtcctatcatctaactttttgaatcactgtattctc |
| CT620 | For | ggggacaagtttgtacaaaaaagcaggcttggaaggagatagaaccatgggttctatgaacatatttaataaaattaactctg |
|  | Rev | ggggaccactttgtacaagaaagctgggtcttactaaccagccagttttcttgtt |
| CT621 | For | ggggacaagtttgtacaaaaaagcaggcttggaaggagatagaaccatggaccgtattcatcgtacacaagg |
|  | Rev | ggggaccactttgtacaagaaagctgggtcttactatcttaagagattacgcgctaat |
| CT621_Cter_ | For | ggggacaagtttgtacaaaaaagcaggcttggaaggagatagaaccatgggtttgagtaataactttgttttgac |
| (DUF582_CT621_) | Rev | ggggaccactttgtacaagaaagctgggtcttactatcttaagagattacgcgctaat |
| CT711 | For | ggggacaagtttgtacaaaaaagcaggcttggaaggagatagaaccatggcaatacaacctacatccatttcttta |
|  | Rev | ggggaccactttgtacaagaaagctgggtcctattatttaaatctacggatcaacttagc |
| CT712 | For | ggggacaagtttgtacaaaaaagcaggcttggaaggagatagaaccatgggaaaccatccgattcca |
|  | Rev | ggggaccactttgtacaagaaagctgggtcttactagctagaagccaatgttct |
| CT671 | For | ggggacaagtttgtacaaaaaagcaggcttggaaggagatagaaccatggaattaaataaaact |
|  | Rev | ggggaccactttgtacaagaaagctgggtcctattatatatgagcttcttc |
